# The efficacy of augmented reality exposure therapy in the treatment of spider phobia—a randomized controlled trial

**DOI:** 10.3389/fpsyg.2024.1214125

**Published:** 2024-02-19

**Authors:** Tomas Jurcik, Svetlana Zaremba-Pike, Vladimir Kosonogov, Abdul-Raheem Mohammed, Yulia Krasavtseva, Tadamasa Sawada, Irina Samarina, Nilufar Buranova, Peter Adu, Nikita Sergeev, Andrei Skuratov, Anastasia Demchenko, Yakov Kochetkov

**Affiliations:** ^1^School of Psychology, HSE University, Moscow, Russia; ^2^HSE University, Moscow, Russia; ^3^Department of Social and Behavioral Change, School of Public Health, University for Development Studies, Tamale, Ghana; ^4^Department of Pedagogy and Medical Psychology, Sechenov University, Moscow, Russia; ^5^Department of Psychology, Lomonosov MSU, Moscow, Russia; ^6^Center for Cognitive Therapy, Moscow, Russia; ^7^Department of Psychology, Russian-Armenian (Slavonic) University, Yerevan, Armenia; ^8^Akian College of Science and Engineering, American University of Armenia, Yerevan, Armenia; ^9^European University of Armenia, Yerevan, Armenia; ^10^Department of Healthcare, P. B. Gannushkin Moscow Clinical Psychiatric Hospital No. 4, Moscow, Russia; ^11^Wellington Faculty of Health, Victoria University of Wellington, Wellington, New Zealand; ^12^Department of Computer Engineering, HSE University, Moscow, Russia

**Keywords:** augmented reality, *in-vivo*, exposure therapy, phobia, spider

## Abstract

The evidence for the use of Augmented Reality (AR) in treating specific phobias has been growing. However, issues of accessibility persist, especially in developing countries. The current study examined a novel, but relatively simple therapist guided smartphone-based AR Exposure Treatment (ARET) of spider phobia. Participants who reported symptoms of Arachnophobia were randomized into one of three comparison groups: ARET (*n* = 20), traditional *in vivo* exposure therapy (IVET; *n* = 18) and a waitlist control group (*n* = 17). Behavioral approach, subjective symptom measures, and galvanic skin response were assessed pre- and post-treatment. The study was concluded with a one-month follow up assessment. Results indicated that both treatment groups showed statistically significant and clinically meaningful improvements in behavioral approach at post-test that were maintained at 1 month follow- up, compared to the wait-listed group. Moreover, the treatment groups demonstrated significant improvements in subjective symptom report at 1-month follow up. Given its utility and potential accessibility, our findings suggest that future AR evaluation research could be conducted in therapy settings with minimal resources.

## Highlights

- We developed and tested a therapist guided Smartphone App for Augmented Reality Exposure Therapy (ARET) of Spider Phobia in a middle-income country.
- We completed a Randomized Control Trial comparing ARET to *In Vivo* Exposure Therapy (IVET) and a waitlist control group.
- ARET and IVET produced similar symptom and behavior approach improvements compared to the control group.
- ARET may be an efficacious treatment in settings with limited resources.

## 1 Introduction

Anxiety disorders are a group of debilitating conditions that affect a substantial proportion of the world’s population, and represent some of the most common mental disorders (Bandelow and Michaelis, 2015). While there are numerous types of anxiety disorders such as generalized anxiety disorder, panic disorder, social anxiety, and specific phobias, many are effectively treated with some form of exposure therapy (Kaplan and Tolin, 2011). For Specific Phobias (i.e., the topic of the current paper) effective treatments typically involve having the patient confront the feared object for a sufficiently long period of time in order to facilitate habituation and for patients to develop new and more benign associations with the object. Thus, patients benefit from inhibitory learning as they realize the feared outcome does not occur upon encountering the stimulus (Craske et al., 2014). Simply put, the patient approaches and extensively interacts with the object, whether animate or inanimate, in the absence of the feared outcome, such as being harmed (see Foa and Kozak, 1986). Contemporary phobia treatments often involve *in vivo* or imaginal exposures, although more recently modern treatments have included interventions involving Information Technology (IT) with some success; that is using Virtual (VR) or Augmented Reality (AR). However, many of these enhanced treatments involve costly systems including computers that project a graphical representation of the feared object onto bulky head mounted displays and sometimes include other complex electronic appendages such as sensors (e.g., Figure 3 in Baus and Bouchard, 2014). In the current study, we examined whether a simple guided AR program (Sergeev and Skuratov, 2018, see also AR device) run on a smartphone that simultaneously functions as a head-mounted display can efficiently treat arachnophobia (fear of spiders) symptoms by projecting a graphic image of a moving spider superimposed on real objects in the environment. Such basic programs and devices, if shown to be effective, could simplify treatments of specific phobias and be especially useful to clinics, therapists, and clients in cost-conscious settings, such as those found in developing countries.

## 2 Specific phobias

According to the Diagnostic and Statistical Manual of Mental Disorders (5th ed.; DSM-5; American Psychiatric Association, 2013), Specific Phobias are characterized by intense fear related to a specific object or situation, and a strong desire to avoid either the situation or object. Significant anticipatory anxiety and avoidance behavior often prevents or limits confrontations with the phobic stimulus. Symptoms must persist for at least 6 months, interfere with the normal activities of daily living, and cannot be explained by other mental illnesses.

The DSM-5 (American Psychiatric Association, 2013) outlines five different types of specific phobias: natural environment phobias (e.g., storms, heights etc.), blood-injection-injury phobias, situational phobias (e.g., flying, elevators), animal phobias (e.g., dogs, spiders), and others that do not fit into any of the four types (e.g., vomiting). Data from 22 countries revealed that the lifetime prevalence rate for specific phobia was 7.4%; gender differences suggest that females have a higher (9.8%) prevalence rate than men (4.9%) (Wardenaar et al., 2017). Animal phobias make up one of the most common forms of specific phobia (Essau et al., 2000). For the purposes of this study, we will focus on spider phobia, a type of animal or insect phobia.

Arachnophobia, or fear of spiders, presents one of the many animal phobias (American Psychiatric Association, 2013) and is endemic to a sizable group of people within the population. For instance, Fredrikson et al. (1996) observed a point prevalence rate of 3.5%. Like other small animal phobias, spider phobia is found to impair the psychological health and overall quality of life of the affected persons (Muris and Merckelbach, 1998).

The majority of those affected by the disorder adjust their lives accordingly (i.e., using avoidance strategies) instead of seeking treatment (Bandelow and Michaelis, 2015). This indifference or aversion to treatment seeking may be partly due to stigma or simply due to the fact that the phobic object can oftentimes be easily avoided. Nonetheless, spiders are relatively common even in more northern latitudes, and those with arachnophobia can be significantly impaired especially if they engage in avoidant behavior that can lead to maladaptive outcomes (e.g., fearing to enter the cellar or garage to work on something important, or rapid escape behavior that may endanger themselves or others in the process). Fortunately, effective treatments are available. In this regard, a meta-analysis of randomized clinical studies has shown exposure-based therapy, especially those utilizing *in vivo* contact, to be the most effective treatment for small animal phobias such as arachnophobia (Wolitzky-Taylor et al., 2008).

## 3 Treatments

*In Vivo* Exposure Therapy (IVET) involves a therapist guided systematic exposure to a real (usually live) animal in the hopes of getting the patient with the phobia to overcome the fear of the stimulus through a process of habituation (Foa et al., 2007; Wolitzky-Taylor et al., 2008). The assumption is that the fear reduction will generalize to similar stimuli. While this treatment is widely accepted among clinicians, studies have shown patients’ preference for it is limited. About 25% of patients refuse to get enrolled into IVET or drop out of treatment (Garcia-Palacios et al., 2007). A viable explanation for the high attrition rate owes to the disquieting sense of having to face the feared animal (Gunter and Whittal, 2010; Meuret et al., 2012). Although exposing patients to their fears might constitute an effective treatment of overcoming such fears, it is important that patients be treated for their condition humanely and ethically. That is, patients should have a choice in selecting their preferred treatment. Two main effective approaches are flooding and graduated exposure; clients and therapists often find the latter to be less stressful as it involves gradually exposing the client to the feared object along a hierarchy (e.g., viewing a photo of the stimulus, then viewing the stimuli from a distance, before systematically approaching it, and eventually touching it) instead of exposing the client to the object at close range from the beginning (e.g., Schumacher et al., 2015). It is in this spirit that “flooding”—a type of exposure therapy once considered a gold standard in the field, has now been discontinued in numerous settings (Gunter and Whittal, 2010; Deacon, 2012). Graded variations of exposure therapy are thus generally preferred in contemporary clinical contexts (e.g., Gilroy et al., 2000; Gotestam and Hokstad, 2002).

Access to the phobic stimulus is not always feasible in the clinic, and thus exposure without live stimuli has also been implemented. Such exposure might be completed using symbolic stimuli (i.e., through pictures) or in imagination (e.g., imaginal exposure using systematic desensitization; Gotestam and Hokstad, 2002). However, more recently treatment approaches have also leveraged advances in IT for diagnosis and treatment of clinical conditions. For specific phobias, the use of Virtual Reality Exposure Therapy (VRET) and Augmented Reality (AR) devices have become increasingly popular.

IT assisted treatments are becoming increasingly widespread (Adu et al., 2021). Although some patients and their psychotherapists were previously more reticent in using IT, in recent years the use of online and other computer-based technologies in psychotherapy appear to be becoming increasingly common in treating and preventing the recurrence of various mental disorders. This trend may have been accelerated following the lockdowns and other government restrictions surrounding the COVID-19 pandemic (Jurcik et al., 2020; Adu et al., 2021).

VRET, like IVET, utilizes the therapeutic process of gradual exposure to a phobic animal. However, VRET substitutes interaction with a live animal in a real physical environment with a digital version in a virtual environment, thereby creating an immersive effect. VRET can induce fear (Baus and Bouchard, 2014), without the logistical requirements of having a real animal present in the room. A high acceptance rate reported by patients in favor of VRET attests to the preference for this approach (Garcia-Palacios et al., 2007). VRET is an effective approach in reducing patients’ phobic anxiety, thus improving their ability to interact with the feared animal (Botella et al., 2017); clinical trials indicate the efficacy of VRET compared to waitlist control groups in treating spider phobia (Garcia-Palacios et al., 2002; Hoffman et al., 2003). Thus, VR technology can circumvent issues that come with traditional *in vivo* exposure therapy such as control over the animal stimuli and scenario. AR, a more recent variant of VR, presents an even more enriched exposure experience, and arguably offers greater ecological validity.

Augmented Reality Exposure Therapy (ARET) allows for an interaction with a virtual phobic stimulus in a real physical environment, permitting the use of the patient’s own body in the interaction. This means that, unlike virtual reality, AR has the advantage of merging virtual elements with the real world whereas patients are immersed in a fully synthetic environment in virtual reality.

Evidence supporting the efficacy of ARET has been encouraging regarding the treatment of specific phobias in recent years. Earlier pilot studies had hinted at the potential efficacy and effectiveness of ARET in treating cockroach phobia, even at one year follow-up period (Botella et al., 2010), while prolonged exposure of participants to virtual spiders and cockroaches significantly reduced fear and avoidance of these (Juan et al., 2005). Botella et al. (2016) conducted a larger treatment study for phobia for cockroaches and spiders using both IVET (*n* = 31) and ARET (AR 5DT head-mounted display) (*n* = 32) in a randomized controlled trial using one-session treatment therapy guidelines. The study revealed that participants in the ARET and IVET conditions improved significantly on all outcome measures at post-treatment and follow-up. All measures indicated large effect sizes (*d* > 0.8) in both conditions. Finally, researchers reported that acceptance levels for ARET tend to be higher compared with IVET. All of these studies also used the one-session treatment guidelines developed by by Öst et al. (1991), which were also followed in our study, described below.

More recently, an aggregated three study comparison explored the treatment efficacy of VRET, ARET, and IVET approaches for small animal phobia, and showed ARET to be comparable in efficacy to the other two treatments (Suso-Ribera et al., 2019). Moreover, both VRET and ARET were shown to be as efficacious as IVET, regardless of the level of baseline anxiety, although there were slight advantages for the IVET condition with regards to distance covered for those with poorer approach behavior prior to treatment (Suso-Ribera et al., 2019). Especially relevant to the current study, an AR application (app) was found to be beneficial for both subclinical and clinical fear of spiders as researchers reported that repeated home-use of a standalone, smartphone-based, gamified AR exposure app was effective in reducing (subjective) spider phobia in the intervention group (*n* = 33) compared with the control group (*n* = 33) (Zimmer et al., 2021). Hence, ARET is a promising treatment approach that deserves further study, especially using smartphone technology.

The extant literature depicts the promising nature of ARET in treating specific phobias, however, certain limitations exist such as the lack of rigorous controls and measurements. For instance, efficacy studies may include treatment conditions consisting of ARET and IVET but lack a waitlist control group (e.g., Botella et al., 2016). A control group would have helped more accurately gage pre- to post-treatment response vis-à-vis regression to the mean effects. In addition, physiological measures besides self-report could have been employed to support the significance of the latter (see also Botella et al., 2010; Baus and Bouchard, 2014). Zimmer et al. (2021), employed a control group, but did not include a physiological outcome measure, and did not include an IVET condition in the study’s design. IVET is a clinically accepted treatment and thus ideally could be part of the treatment conditions to more accurately ascertain the effect of ARET. Thus, we conducted a controlled head-to-head comparison of both treatments using traditional subjective measures, as well as objective behavioral approach and a physiological indicator—skin conductance response (SCR)—to assess anxiety or fear via sweat gland activity (see Spence, 2018).

Moreover, existing research in this area, largely conducted in the West, may not apply directly to non-Western contexts due to cultural variations in spider beliefs and potential differences in spider phobia prevalence (Prokop et al., 2010; Eaton et al., 2018). More generally, there are also varying levels of acceptance and engagement in psychological treatments influenced by factors such as stigma (Kirmayer, 2007; Adu et al., 2021). For instance, lower trust and endorsement of professional help-seeking have been reported in Russian compared to Western settings (e.g., Nersessova et al., 2019; Cavanagh et al., 2022). With regards to arachnids, Boyko (2020) noted that spiders are perceived similarly but also in some different ways in Russian compared to English and French folklore contexts. For instance, there appear to be more positive adages regarding spiders in these Western cultural-linguistic traditions (e.g., good news, luck, and guests) that may be absent in the Russian one. Finally, given the technological focus of the current study, we recognize that a large proportion of Russians use smartphones (over 70%) but this percentage is still less than the proportion found in the United States or Germany (over 80%; see Statista, 2023). Hence, the relevance or benefits of a gamified smartphone technology should not be assumed to be equal in different regions of the World. Indeed, earlier literature emphasized the need to conduct more clinical and adaptation research to examine the acceptability and generalizability of Western-developed interventions in Russia (see Jurcik et al., 2013). The current study provides such evidence for the treatment of spider-focused anxiety.

Accordingly, the current study aimed to test the efficacy of ARET in a brief treatment of spider phobia using a Randomized Control Trial (RCT) employing multiple treatment conditions (ARET, IVET, control) and multiple outcome measures (self-report and physiological measures) in a non-Western (Russian) context. To the best of our knowledge, the current RCT design overlaps to some extent with earlier research, but it is also unique in that it uses a novel therapist guided phone app designed for the study, and compares this intervention to both an established treatment (IVET) *and* a waitlist control group, monitoring gains up to one month follow up.

## 4 Hypotheses

Based on our review of the literature, we hypothesized that the two exposure treatments (IVET and ARET) would yield comparable and significant benefits in reducing the fear of spiders as measured by symptom reduction, galvanic skin response, and approach behavior, and that this outcome would be sustained at the 1-month follow up assessment. In turn, we hypothesized that both exposure groups would show significant improvements above and beyond the waitlist control group on these outcome measures.

## 5 Method

### 5.1 Participants

**Figure 1:**
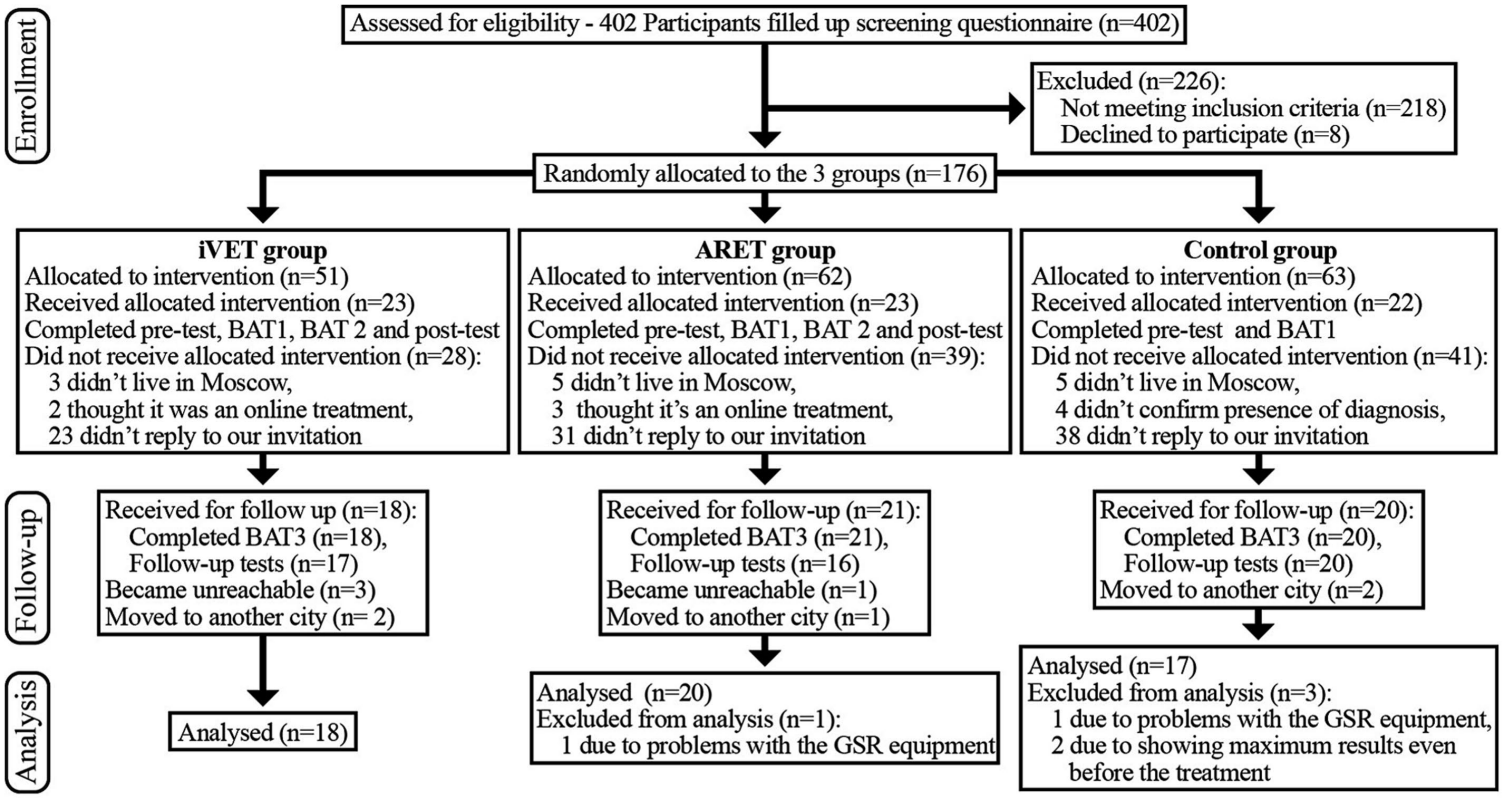
Consort flow diagram.

A recent meta-analysis (Carl et al., 2019) showed a large effect size for VRET vs. waitlist (*g* = 0.90). We thus estimated that we would require 12 participants per group to conduct within group comparisons or 21 participants per group for between group comparisons (1 − β = 0.80). However, we also calculated *post hoc* power for each comparison to check whether we reached the threshold of 1 − β = 0.80. Four hundred and two people were originally screened for the study, but only 176 of them met selection criteria (described below); these potential participants were invited to the study and randomly assigned into one of three groups: (1) AR Exposure Treatment (*n* = 62), (2) *in vivo* exposure treatment (*n* = 51), and (3) waitlist control (*n* = 63). A substantial proportion (67%) did not follow through with the invitation, due to various reasons (see Figure 1) and the final sample yielded 55 participants who completed the study and follow up; they were aged from 18 to 46 years (*M*_age_ = 30.98; *SD*_age_ = 6.06), of which 96% were female. The *in vivo* exposure group (*n* = 18; *M*_age_ = 30.59; *SD*_age_ = 6.08), AR treatment group (*n* = 20; *M*_age_ = 31.58; *SD*_age_ = 5.57), and waitlist group (*n* = 17; *M*_age_ = 30.71; *SD*_age_ = 6.82) were similarly sized. We did not find any significant age or gender differences between the three groups, nor did we find any evidence of selective attrition. All participants were Russian-speakers living in Moscow or the surrounding region. See Figure 1 flow diagram for how this final group of participants was obtained.

### 5.2 Design

We completed a Randomized Controlled Trial (RCT). Participants were randomly assigned using a computer program, into one of three comparison groups: an AR treatment group, an *in vivo* treatment group, and a waitlist control group. Differences between groups were examined for reported symptoms and behavioral avoidance from pre- to post-treatment (see Measures below). The final group of participants who completed the study are described below.

### 5.3 Procedure

The study was approved by the institutional review board. The majority of participants were recruited through online social media platforms (Instagram); snowball sampling techniques were also employed (via Facebook). The study included six stages: (1) participant recruitment and initial screening procedures; (2) randomization into three groups and invitation; (3) pre-treatment symptom assessment, (4) exposure therapy or waitlist, (5) post-treatment assessment and (6) reassessment at 1 month follow-up.

#### 5.3.1 Online screening and randomization

An online screening survey was used to check for the eligibility of potential participants according to Diagnostic and Statistical Manual, 5th edition (DSM-5, American Psychiatric Association, 2013; Zimmerman, 2013) criteria for Specific Phobia (i.e., fear of spiders). One of the authors (SZP) enrolled participants and excluded those with psychotic disorders or substance use disorder, heart diseases, and those that did not meet the DSM-5 criteria. The online screening also asked participants’ availability to be involved in the study in the following 2 months. One hundred seventy-six respondents met screening criteria and expressed their consent to participate in the study. They were randomized using blocked randomization (using randomizer.org by co-author SZP) over six successive waves (i.e., as new respondents joined the study and underwent the selection process) into one of three different groups. Of 176 participants assigned for intervention (by co-authors SZP, YK, NB, IS), 108 participants dropped out from the study due to various reasons.

As a result, 68 participants took part in the RCT experiment, and 57 of these took part in the follow up part of the study (1 month after the first visit). We faced technical problems with the SCR recording for two participants and excluded them. Thus, our final analyses were based on 55 participants. To clarify the selection process, please refer to the Consort graph of the study (see Figure 1). The groups did not differ in the proportion of drop-outs, *χ*^2^ = 0.58, *p* = 0.75.

#### 5.3.2 Pre-test

Table 1 indicates the order of measures for the different phases of the study. Before visiting the University testing location, 68 participants filled a battery of tests (see Table 1), used in the analyses as pre-test data. At the University testing location an additional screening interview was performed (in addition to the first online screening, above) to confirm that the participants met inclusion and exclusion criteria for Specific phobia during participants’ selection process using DSM–5 criteria (American Psychiatric Association, 2013). The study included participants who met the criteria of Specific Phobia Diagnosis (300.29; interview items were translated from Zimmerman, 2013). The participants gave their consent to participate in the study.

**Table 1:** Order of measures in different phases of the study.

Phase of the study	Questionnaire names
*Online Screening:* All participants	APA questions for specific phobia, PHQ, FSQ
*Pre-test:* IVET, ARET, WL	SPQ, GAD-7, BBQ, CEQ, Screening interview with APA questions for specific phobia, BAT, SCR
*Treatment:* IVET, ARET	SCR
*Post-test:* ARET, IVET	SPQ, FSQ, GAD-7, PHQ, BBQ, SSQ*, IPQ*, SUS*, BAT, SCR
*1 month follow up test:* IVET, ARET, WL	SPQ, FSQ, GAD-7, PHQ, BAT, SCR

PHQ, Patient Health Questionnaire; FSQ, Fear of Spiders Questionnaire; GAD-7, General Anxiety disorder; BBQ, The Brunnsviken Brief Quality of Life Inventory; CEQ, Credibility Expectancy Questionnaire; SSQ, Simulator Sickness Questionnaire; IPQ, iGroup Presence Questionnaire; SUS, The System Usability Scale treatment; SCR, Skin Conductance Response. *Only used in ARET group (see Supplementary material).

Following the screening interview, all participants underwent the Behavioral Avoidance Test (BAT, described below). Next, participants in the *in vivo* group received traditional exposure therapy, while participants from the AR group received exposure therapy with a virtual spider via augmented reality glasses. The Control Group completed the BAT without exposure therapy. During BAT and exposure therapy skin conductance was measured. After the treatment, participants completed questionnaires to assess perceived fear of spiders (SPQ, FSQ), general anxiety level (GAD-7), depression symptoms (PHQ), participants’ impression of the study and other treatment acceptability measures (see Table 1).

#### 5.3.3 Treatment

Participants from the AR group and the *in Vivo* group received exposure therapy, which is a broadly used and evidence-based approach in treating phobias (Öst et al., 1991; Gotestam and Hokstad, 2002; Michaliszyn et al., 2010). However, to facilitate equivalent conditions for both treatment groups (AR and *in vivo*), exposure therapy was slightly adjusted. As such, during ordinary treatment, a patient makes unregulated steps toward the feared object as their anxiety becomes more tolerable; in this experiment, in contrast, the spider is placed closer to the participant. Thus, the adult spider (tarantula breed) was put in a closed transparent plastic jar and was placed in four precisely set distances based on the program’s limitations for where the virtual spider could appear: at 2.5 meters, 1.5 meters, on a table next to the participant and on the participant’s knees (Sergeev and Skuratov, 2018, see AR device in Method). Accordingly, the same distance was set for both treatment groups. A detailed description on how the treatment was conducted is described below.

The exposure consisted of four stages. At the first stage, after the participant was asked to open their eyes, a tarantula spider in a closed transparent plastic jar was demonstrated at a distance of 2.5 meters. At that point the therapist asked the participant to evaluate their perceived anxiety (fear) level - on a Subjective Units of Distress scale (SUDs) - on a scale ranging from 0 to 10. The participants voiced each time the SUDs decreased by 1 point. The procedure was repeated until the SUDs dropped to 50% from the initial level, e.g., from 10 to 5. Progression to each subsequent step in the exposure hierarchy was dependent on a 50% decrease in SUDs levels from the previous step. Next, the participant was exposed to the phobic stimulus at a distance of 1.5 meters from the participant, then on the table close to the participant and on the participant’s knees. After each step participants were asked to close their eyes. After placing the spider to a closer distance, the therapist would ask the participant to open their eyes. This procedure enables clearer measures on the Skin Conductance Response (SCR) device, which was used during all the stages of the experiment. Finally, the stimulus was placed on the participant’s knees at the same moment as the control psychophysiological measure was taken. The same procedures were applied during exposure therapy using the AR device.

#### 5.3.4 Post-test and 1 month follow up

The experiment was followed by the BAT to evaluate improvements in approach post-intervention, and participants completed post-test symptom measures. Debriefing was also provided. The majority of participants reported positive improvements after the exposure therapy, except for one participant from the IVET group, who reported feeling upset by the treatment procedure. This, along with all other participants, were provided with contact information and resources for additional treatment and support. Follow-up took place approximately 1 month after the treatment phase: IVET, ARET, and control groups were invited to repeat the BAT and complete symptom measures again (Table 1). All waitlisted participants who wished to receive treatment after the study received IVET group therapy organized by a clinic director and co-author (YK).

### 5.4 Measures

#### 5.4.1 Behavioral approach test

In the traditional BAT, participants are asked to take increasingly more involved steps toward the feared object starting with entering the room where the object is located and gradually progressing toward holding the feared object for several seconds (Cochrane et al., 2008). The BAT was modified for this study to accommodate for physiological measurement. Instead of the participant approaching the spider, the BAT for this study started with the therapist holding a clear jar containing the spider across the room from the participant. The latter was seated in a chair with skin conductance monitoring measures attached. The participant was asked to close their eyes. The therapist stood at the end or room (5 m from the participant) and the participant was asked to open their eyes. After a 6-s pause—needed to record the skin conductance in response to the spider appearance—the therapist began to gradually approach the participant with the spider in a jar. Participants were explicitly instructed not to close their eyes and try to be in the moment. The BAT stopped as soon as the participant indicated verbally that the therapist should not approach any further (e.g., said “stop”) or take another next step in the BAT (e.g., opening the jar containing the spider). The procedure was fully standardized for all the three groups of the study. The point at which the therapist stopped with the jar during BAT was also noted by the psychophysiologist (co-author VK).

#### 5.4.2 Skin conductance

ActiChamp amplifier (Brain Products, Germany) was used to collect (at 1,000 Hz), amplify, and filter skin conductance. Bipolar Ag/AgCl surface electrodes were placed on the second and fourth fingers of each participant’s left hand. There was a calibration of the resulting raw signal in order to detect an activity within 0–100 microSiemens range. Following Benedek and Kaernbach (2010), in Ledalab 3.2.2, we conducted the continuous decomposition analysis. It permitted us to delete the tonic component from the signal and to analyze only the phasic one which is supposed to better reflect the event-related skin response. The magnitude of peaks that appeared between 0.09 s and 4 s in each condition (i.e., during the first 6 s of the still demonstration before the beginning of BAT at pre-test, post-test and 1-month follow up) were computed as the skin conductance response (Venables and Christie, 1980).

#### 5.4.3 Spider phobia questionnaire (SPQ)

Phobia toward spiders was measured with the SPQ, a 31-item self-report validated scale (Klorman et al., 1974). The dichotomous scale is rated on true or false responses. The scores of the scale can range from 0 to 31 with higher scores indicating greater self-reported phobia for spiders. A sample item on the scale is “I avoid going to parks or on camping trips because there may be spiders around.” The Cronbach’s alpha of the scale in the current was good (mean *α* = 0.74).

#### 5.4.4 Fear of spider questionnaire (FSQ)

The FSQ is an 18-item self-report questionnaire that assesses an individual’s fear toward spiders (Szymanski and O'Donohue, 1995). The items in the scale were constructed to cover several domains of human functioning: cognition, negative attitudes, physiology, behavior, and fear of harm by spiders. A sample item from this scale is: “I now think a lot about spiders”. The scale was rated on seven points ranging from: 1 (not all) to 7 (very much). In the current study, the scale exhibited an excellent internal consistency (mean *α* = 0.90).

#### 5.4.5 General anxiety scale (GAD-7)

Overall anxiety severity was measured with the GAD-7 (Williams, 2014). This scale contains 7 items that measure recent anxiety over the past 2 weeks. A sample item includes “Not being able to stop or control worrying”. The instrument is rated on a 4-point Likert scale ranging from 1 = not difficult at all to 4 = extremely difficult. The internal consistency for the scale in the current study was very good (mean *α* = 0.85).

#### 5.4.6 Patient health questionnaire (PHQ)

The presence of depression severity was measured by the PHQ-9, which contains 9 items rated on a 4-point scale (1 = not at all; 4 = nearly every day) (Kroenke et al., 2001). The instrument is used to screen adult patients at primary care settings for the presence of depression severity. Sample items on the scale include: “Feeling down, depressed, or hopeless” and “Feeling tired or having little energy.” The internal consistency for the instrument in the current study was acceptable (mean *α* = 0.76).

#### 5.4.7 The Brunnsviken brief quality of life scale (BBQ)

The BBQ is a 12-item instrument measuring subjective quality of life (Lindner et al., 2016). The scale measures life quality covering six life domains: creativity, philosophy of life, learning, self-regard, friendships, and recreation. Responses were scored on a 4-point Likert scale (1 = strongly disagree to 4 = strongly agree). “My leisure time is important for my quality of life” and “How I view my life is important for my quality of life” are sample items on the scale. The internal consistency for the scale in the current study was acceptable (mean *α* = 0.78).

#### 5.4.8 Credibility expectancy questionnaire (CEQ)

Treatment expectancy and credibility was measured using the CEQ, one of the most widely used measures of treatment expectancy and rationale credibility in clinical research (Devilly and Borkovec, 2000). A sample item on the scale is “How logical does the therapy offered to you seem” (credibility subscale) or “How much improvement in your symptoms do you think will occur” (expectancy subscale). The measure consists of three items measuring credibility on a 9-point scale (1 = not at all; 9 = very credible) and three items measuring expectancy (two from 0 to 100% and one on a 9-point scale). The scale yielded very good to excellent (*α* = 0.83 for credibility and standardized *α* = 0.89 for expectancy) internal consistency in the current study.

### 5.5 AR device

**Figure 2:**
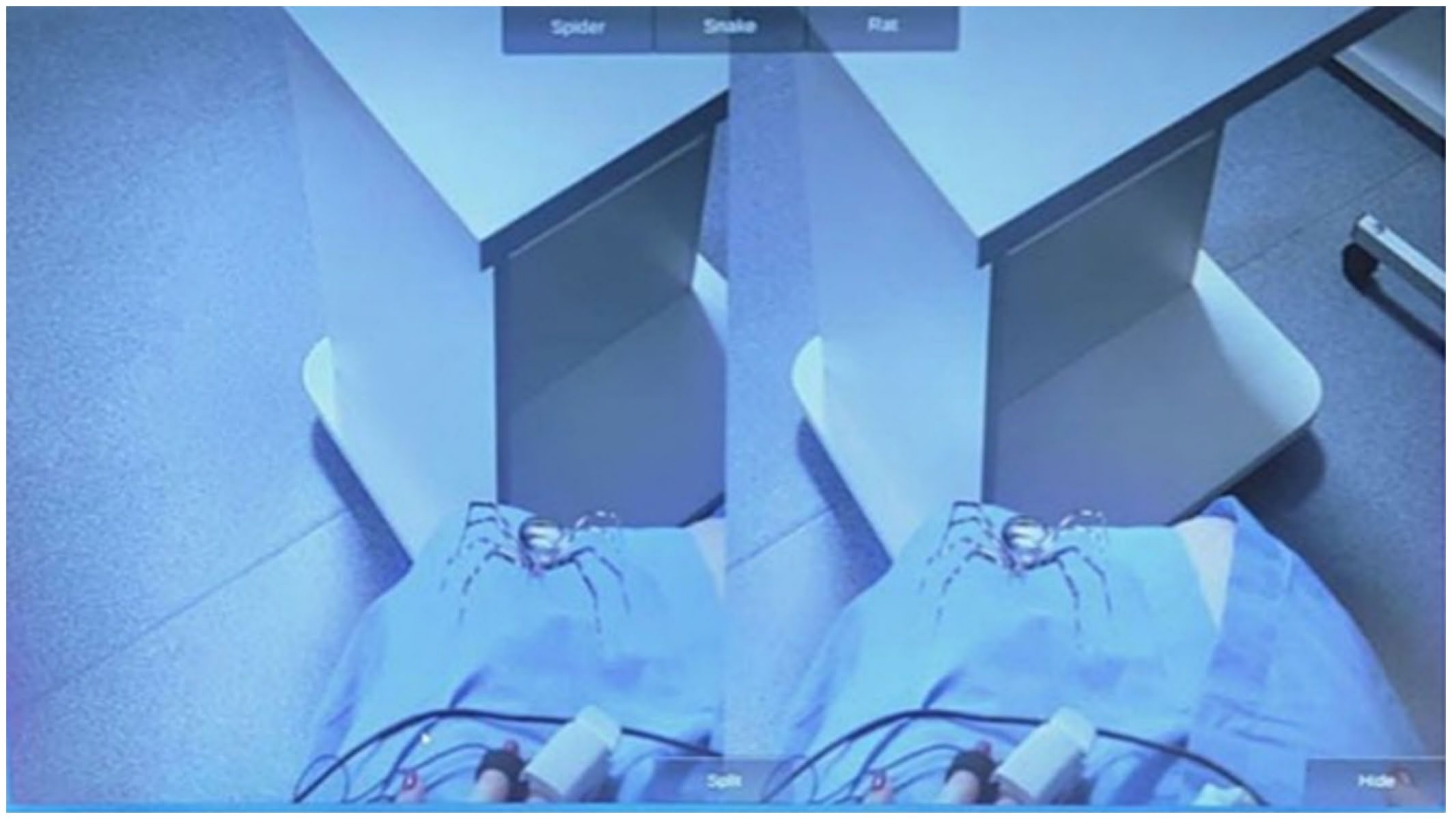
A screenshot of the AR app used in this study depicting spider on the knees of a participant.

The AR device used in this study showed a 3D computer-graphics (3DCG) image of a spider superimposed on a photographic image of a scene “out there” (Figure 2). The device was a head-mounted display that was composed of a smartphone (Lenovo Phab 2 Pro) mounted within a wearable stereoscope (VR headset). It was equipped with a photo camera for capturing a photographic image of the scene and with a 3D scanner for capturing a depth distribution of the scene (see Supplementary material for more details). A special Android app developed for this study was installed on the phone. This app recovered the 3D surfaces in the scene based on a depth distribution. Thereafter, a 3DCG image of a spider was superimposed on the photographic image, making the spider appear to be on the surface in the scene. The spider used in the AR app was modeled after the female wasp spider (*Argiope bruennichi*), common to Russia. Participants wore a headset and saw the image of the spider on the LCD screen of the phone. The program of this AR app was uploaded to GitHub.^1^ See Supplementary material for additional AR generated images.

### 5.6 Data preparation and analysis

To deal with any missing item values within questionnaires, we applied multivariate imputation by chained equations (Van Buuren and Groothuis-Oudshoorn, 2011; 26 values or 0.3% were imputed). For cases which dropped out prior to follow up, we used the Last Observation Carried Forward (LOCF) approach in imputing missing scores (European Medicines Agency, 2011). Given the lack of differential attrition rates in our groups, and the possibility of limited symptom remission over time (see Eaton et al., 2018), we decided that this approach would not overly bias our study results while helping us retain power. The skewness and kurtosis for the questionnaires and BAT were between −2 and +2, thus considered to be normally distributed (Kline, 2011) and were not transformed. SCR data were logarithmized (Boucsein, 2012), hence parametric tests were applied for the data analysis.

To assess the effectiveness of the intervention, a series of analyses was carried out. First, one-way ANOVA was used to test the difference between the three groups at pre-test. After the intervention (post-test), a paired-samples *t*-test was carried out in both ARET and IVET groups to understand if both interventions were effective. The final round of analyses involved repeated measures ANOVA where the one-month and post-test assessment was compared with the pre-test assessment in all three groups. Corrections for multiple comparisons were carried controlling the false discovery rate (Benjamini and Hochberg, 1995). BAT and SCR were the objective outcome measures, whereas the questionnaires were the subjective outcome measures. Analyses were carried out using Statistica (StatSoft) and JASP version 0.17.2.1 (JASP Team, 2022).

## 6 Results

### 6.1 Descriptive statistics

The summary statistics of all available measures are provided in Table 2, including those at pre-test, post-intervention, and at 1 month follow-up for the different groups.

**Table 2:** Summary of descriptive statistics for the measures by group and time point.

		ARET		IVET		WL	
		*M*	*SD*	*M*	*SD*	*M*	*SD*
Pre-test	BBQ*	36.95	6.32	39.41	5.37	35.00	7.41
	PHQ	5.40	4.08	4.47	2.92	5.11	3.28
	GAD	5.42	3.67	4.82	2.94	4.24	3.47
	SPQ	20.05	2.72	20.24	3.01	19.58	3.76
	FSQ	91.00	13.90	90.17	14.28	94.00	19.07
	BAT	4.55	1.85	5.28	2.47	4.71	2.34
	SCR	0.84	0.71	1.30	1.05	1.54	1.40
Post-test	PHQ	5.32	3.16	5.72	4.42	–	–
	GAD	5.15	3.98	5.44	4.30	–	–
	SPQ	17.65	5.61	16.94	6.31	–	–
	FSQ	73.95	20.29	73.72	25.32	–	–
	BAT	7.10	2.79	8.28	1.71	–	–
	SCR	0.80	1.00	0.65	1.01	–	–
	IPQ*	−1.74	10.18	–	–	–	–
	SUS*	73.64	12.88	–	–	–	–
	SSQ*	5.89	5.49	–	–	–	–
Follow-up	PHQ	5.47	3.49	6.28	4.18	5.94	3.90
	GAD	4.47	3.61	7.50	4.87	6.18	4.57
	SPQ	15.42	5.85	13.72	5.63	20.18	4.10
	FSQ	70.21	20.99	67.33	20.80	85.00	24.02
	BAT	8.10	3.21	8.67	1.94	5.94	2.95
	SCR	0.20	0.26	0.50	0.72	0.45	0.46

M, Mean; SD, Standard deviation; PHQ, Patient Health Questionnaire; FSQ, Fear of Spiders Questionnaire; GAD-7, General Anxiety disorder; BBQ, The Brunnsviken Brief Quality of Life Inventory; CEQ, Credibility Expectancy Questionnaire; SSQ, Simulator Sickness Questionnaire; IPQ, iGroup Presence Questionnaire; SUS, The System Usability Scale treatment; SCR, Skin Conductance Response. *See Supplementary material.

### 6.2 Pretest check between groups

Demographics such as age, *F*(2, 52) = 0.14, *p* = 0.87, and gender, *χ*^2^ (1, *N* = 55) = 0.02, *p* = 0.98, did not differ between the three groups at baseline assessment. There was no significant mean difference among the three groups on quality of life inventory, BBQ, *F*(2, 50) = 2.02, *p* = 0.14, and somatic symptoms (PHQ), *F*(2, 51) = 0.33, *p* = 0.72. Other symptom scales did not reveal baseline differences between groups: SPQ, *F*(2, 50) = 0.19, *p* = 0.83; GAD, *F*(2, 50) = 0.550, *p* = 0.58); FSQ, *F*(2, 52) = 0.29, *p* = 0.75); BAT, *F*(2, 52) = 0.552, *p* = 0.58; SCR, *F*(2, 52) = 2.06, *p* = 0.14).

For the CEQ, the mean difference between the ARET and IVET was not statistically significant for either credibility, *t*(35) = −0.17, *p* = 0.86, *M*_IVET_ = 21.06, *SD*_IVET_ = 4.22, *M*_ARET_ = 20.08, *SD*_ARET_ = 4.76, or expectancy, *t*(34) = 1.23, *p* = 0.23, *M*_IVET_ = 17.05, *SD*_IVET_ = 4.80, *M*_ARET_ = 18.83, *SD*_ARET_ = 3.89, prior to random assignment. That is, both treatment groups reported similar credibility and expectancy prior to intervention.

### 6.3 Objective measures

#### 6.3.1 Behavioral approach over the course of treatment

**Figure 3:**
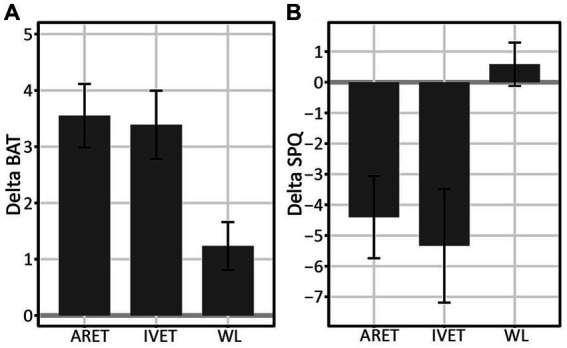
**(A)** Changes in Behavioral Approach (DeltaBAT) between pre-test and one-month follow up (1 m-pre) for Augmented Reality Exposure Therapy (ARET), *In vivo* Exposure Therapy (IVET), and Waitlist Control (WL) groups. **(B)** Changes in SPQ (DeltaSPQ) between pre-test and one-month follow up (1 m-pre) for Augmented Reality Exposure Therapy (ARET), *In Vivo* Exposure Therapy (IVET), and Waitlist Control (WL) groups. Error bars represent standard errors.

An analysis of variance (ANOVA) showed a main effect of group on changes (delta henceforth) in behavioral approach as measured by the BAT (1 month minus pretest), *F*(2, 52) = 5.44, *p* = 0.007, η_p_^2^ = 0.17. *Post-hoc* comparisons revealed that both ARET and IVET increased on the BAT score in comparison to WL, *p* = 0.012, *d* = 1.05, *post hoc* 1-β = 0.87, and *p* = 0.026, *d* = 0.97, *post hoc* 1-β = 0.80, respectively. This pattern is further illustrated in Figure 3A.

On the overall degree of approach behavior for each group (BAT), the ARET group increased from pre-test to 1-month follow up, *t*(19) = 6.29, *p* < 0.001, *d* = 1.41, *post hoc* 1-β > 0.99. The same pattern was found for the IVET, *t*(17) = 5.59, *p* < 0.001, *d* = 1.32, *post hoc* 1-β = 0.99 and WL groups, *t*(16) = 2.91, *p* = 0.010, *d* = 0.71, *post hoc* 1-β = 0.78. More detailed examination of treatment effects over time revealed that BAT in the ARET group increased significantly from pre-test to post-test with large effects, *t*(19) = 4.85, *p* = 0.001, *d* = 1.08, *post hoc* 1-β > 0.99 and continued to increase from post-test to 1-month follow up, *t*(19) = 2.13, *p* = 0.047, *d* = 0.48, *post hoc* 1-β = 0.48. As for the IVET group, BAT increased from pre-test to post-test, *t*(17) = 4.87, *p* = 0.001, *d* = 1.15, *post hoc* 1-β >0.99 and gains were maintained with nonsignificant changes from post-test to 1-month follow up, *t*(17) = 1.28, *p* = 0.22.

On the differences between ARET and IVET at posttest and 1-month follow up assessment, as expected, Delta BAT (post-test vs. pre-test) did not differ between ARET and IVET, *t*(36) = 0.56, *p* = 0.58. Similarly, Delta BAT (1 month follow up vs. post-test) did not differ between ARET and IVET, *t*(36) = 1.07, *p* = 0.29.

#### 6.3.2 Skin conductance response

ANOVA showed no main effect of group for changes in pre-test to 1 month follow-up in SCR (DeltaSCR [1 month-pretest]), *F*(2, 52) = 9.41, *p* = 0.400, η_p_^2^ = 0.035. However, some within group effects were obtained. SCR in the ARET group was found to decrease from pre-test to 1-month follow up, *t*(19) = 4.72, *p* = 0.001, *d* = 1.06, *post hoc* 1-β = 0.98. The same pattern was found for the IVET [*t*(17) = 4.13, *p* = 0.001, *d* = 0.97, *post hoc* 1-β = 0.96] and WL groups [*t*(16) = 3.16, *p* = 0.006, *d* = 0.77, *post hoc* 1-β = 0.91]. Additional *post-hoc* analyses can be found in the Supplementary material.

### 6.4 Subjective symptom measures

#### 6.4.1 Spider phobia questionnaire

ANOVA showed a main effect of group on the SPQ at 1 month follow-up vs. pretest (Figure 3B, DeltaSPQ [1 m − pretest]), *F*(2, 52) = 5.06, *p* = 0.010, η_p_^2^ = 0.16. *Post-hoc* comparisons revealed that both ARET and IVET decreased in SPQ score in comparison to WL (*p* = 0.037, *d* = 0.84, *post hoc* 1-β = 0.89 and *p* = 0.014, *d* = 1.00, *post hoc* 1-β = 0.89 respectively).

Over the course of the study, SPQ in the ARET group was found to decrease from pre-test to 1-month follow up, *t*(19) = 3.34, *p* = 0.004, *d* = 0.77, *post hoc* 1-β = 0.90. The same pattern was found for the IVET group, *t*(17) = 4.47, *p* < 0.001, *d* = 1.01, *post hoc* 1-β = 0.99. However, SPQ scores did not change in the WL group, *t*(16) = 0.84, *p* = 0.42, *d* = 0.20. More specifically, SPQ in the ARET group decreased from pre-test to post-test, *t*(19) = 2.60, *p* = 0.018, *d* = 0.60, *post hoc* 1-β = 0.74, and then did not change from post-test to 1-month follow up, *t*(19) = 1.69, *p* = 0.110, *d* = 0.39. For the IVET group, SPQ decreased from pre-test to post-test, *t*(17) = 2.03, *p* = 0.033, *d* = 0.56 and gains were maintained with a marginally significant improvement from post-test to 1-month follow up, *t*(17) = 2.11, *p* = 0.050, *d* = 0.50. Between treatment group comparisons at various time points were nonsignificant: Changes from pretest to post-intervention (DeltaSPQ [post−pretest]) did not differ between ARET and IVET, *t*(36) = 0.30, *p* = 0.76. Similarly, Delta SPQ (1 month-post-test) did not differ between ARET and IVET, *t*(36) = 0.10, *p* = 0.92. Thus, the overall findings suggest similar gains for the two treatments.

#### 6.4.2 Fear of spiders questionnaire

An analysis of variance showed no main effect of group (condition) on the FSQ over the course of the study, from pre-test to follow-up (DeltaFSQ, 1 m-pre), *F*(2, 52) = 1.97, *p* = 0.150, η_p_^2^ = 0.07. Within each condition, FSQ in the ARET group was found to decrease from pre-test to 1-month follow up, *t*(19) = 3.76, *p* = 0.001, *d* = 0.86, *post hoc* 1-β = 0.93. The same pattern was found for the IVET group, *t*(17) = 3.65, *p* = 0.002, *d* = 0.86, *post hoc* 1-β = 0.93. However, In the WL group, FSQ decreased only marginally, *t*(16) = 2.11, *p* = 0.051, *d* = 0.51, *post hoc* 1-β = 0.51. Additional *post-hoc* analyses can be found in the Supplementary material. Thus, improvements were similar for the two treatment groups, but they did not significantly outperform WL on this measure.

#### 6.4.3 Generalized anxiety disorder

To measure specificity of the treatment effects, participants also completed the GAD-7. An ANOVA showed a main effect of group (condition) from pre-test to follow-up (DeltaGAD, 1 month-pretest), *F*(2, 52) = 6.54, *p* = 0.003, η_p_^2^ = 0.20. *Post-hoc* comparisons revealed that both WL and IVET *increased* on the GAD score in comparison to ARET (*p* = 0.015, *d* = 0.83, *post hoc* 1-β = 0.67 and *p* = 0.001, *d* = 1.13, *post hoc* 1-β = 0.92 respectively). That is, GAD scores in the ARET group remained stable from pre-test to 1-month follow up, *t*(19) = 1.71, *p* = 0.102, *d* = 0.39, but increased for the IVET, *t*(17) = 2.37, *p* = 0.031, *d* = 0.58, *post hoc* 1-β = 0.64 and WL groups, *t*(16) = 2.86, *p* = 0.011, *d* = 0.70, *post hoc* 1-β = 0.77. Additional *post-hoc* analyses can be found in the Supplementary material.

#### 6.4.4 Physical health questionnaire

An ANOVA showed no main effect of the group on the DeltaPHQ (1 month-pretest), *F*(2, 52) =1.90, *p* = 0.160, η_p_^2^ = 0.07. PHQ in the ARET group did not decrease from pre-test to 1-month follow-up, *t*(19) = 0.13, *p* = 0.899, *d* = 0.03, and marginally increased for the IVET group, *t*(17) = 2.10, *p* = 0.052, *d* = 0.51. In the WL group, PHQ also remained relatively stable, *t*(16) = 1.17, *p* = 0.260, *d* = 0.28. Additional *post-hoc* analyses can be found in the Supplementary material.

## 7 Discussion

In the current study, we tested a novel Augmented Reality Exposure Therapy (ARET) for spider phobia using images generated by a program developed for a smartphone device. In a Randomized Controlled Trial (RCT), we compared this novel therapist guided ARET treatment to more traditional *In Vivo* Exposure Therapy (IVET) and Waitlist Control (WLC) groups. Our hypotheses, that the two treatment groups would demonstrate similar outcomes and outperform the waitlist group - in approach and symptom reduction - were partly supported.

Thus, the ARET and IVET groups showed significantly greater approach from pre-treatment to post-treatment and one-month follow-up as measured on the BAT compared to the WLC. The effect sizes were large in both treatment groups (i.e., *d* > 1.0, *post hoc* 1-β > 0.80). We also found a reduction in autonomic nervous system activity as measured by the SCR over time. However, all groups showed similar levels of SCR at one-month follow-up.

For the subjective symptom indicators, we broadly discovered similar patterns of improvements for the treatment groups who outperformed WLC (i.e., SPQ). However, this was not fully corroborated by the FSQ, where there were no significant improvements above WLC, despite overall improvements over time. This discrepancy may have occurred due to our limited power to detect differences, but it also highlights the importance of including a waitlist control condition in head-to-head comparison studies to account for non-treatment related symptom remission (see strengths and limitations section; see also Eysenck, 1952). The non-specific measures (GHQ and PHQ) indicated divergent validity with our phobia specific measures as they showed different patterns (including some increases for some groups) that may have been unrelated to the treatment.

Our current study adds to the extant literature demonstrating the efficacy of augmented and virtual reality treatments in treating spider and other small animal phobias (e.g., Wrzesien et al., 2011, 2015; Botella et al., 2016). Our treatment study findings are consistent with Zimmer et al. (2021) who found significant improvements in behavioral approach in their ARET group compared to WLC. Our study, however, had the benefit of comparing ARET to not only WL, but also traditional IVET. Further, our work also differed from Zimmer in that therapists guided the exposure treatment, thus standardizing the approach, while Zimmer’s participants completed their intervention at home.

Overall, our objective behavioral indicator common to other studies (i.e., BAT) suggested that both ARET and IVET groups enjoyed behaviorally similar improvements. While both treatment groups clearly were superior on the behavioral approach test vis-a-vis the control group following treatment, which was generally corroborated by the SPQ, other symptom and skin conductance level changes did not demonstrate the same clear pattern.

### 7.1 Strengths and limitations

Our current RCT is one of the few studies that utilized a smartphone powered ARET application (cf. Zimmer et al., 2021). It also used a traditional IVET comparison group allowing us to assess any differences between treatments, and utilized a physiological measure (i.e., SCR) in addition to more common indicators (BAT, SPQ). Prior to assignment at least, ARET and IVET participants were not differentially biased, judging the intervention they were to receive as equally credible. We also did not notice differential drop out between the groups and the groups were demographically comparable. This finding suggests that our AR device was similar in acceptability to traditional treatment. Relatedly, other data suggests that the AR device had acceptable usability ratings and generated minimal cybersickness (see Supplementary material).

Our study also represents one of the few published studies on the use of ARET technology in a country that was part of the former Soviet Union. There has been a call in recent years for conducting studies beyond WEIRD settings (i.e., Western, Educated, Industrialized, Rich, and Democratic; Henrich et al., 2010). Our study partly meets this need. Despite some potential cultural and regional differences regarding the perceptions of spiders, stigma related to treatments for mental illness, and smartphone use (see Introduction), our findings are generally consistent with what we would have expected in a Western sample.

However, our study was not without limitations. Our smaller sample may help explain why we failed to find significant group differences on some measures (e.g., skin conductance, FSQ), although the analyses were generally not underpowered and we typically obtained moderate to large effects. Next, although the therapists and participants were not blind to the condition, the treatment procedure was carefully standardized to limit allegiance bias. For instance, the study involved pre-set distances at which the virtual spider would appear. Nevertheless, this made the treatment exposures somewhat artificially rigid, as the participants could not approach the spider in a more finely graded fashion. The spider was assessed by some viewers as not being sufficiently realistic, and it is possible that the AR group may have been more prone to cognitive avoidance and safety strategies (e.g., minimizing the purpose of the graphically generated spider); however, *in vivo* conditions can also be prone to safety behaviors such as avoiding direct visual contact of the phobic object.

Our qualitative observations following the exposure session supported such impressions. That is, some participants reported that they imagined or persuaded themselves that the spider was not real (i.e., that it was just “a toy” in case of IVET or that it was just a computer program, in case of ARET). Nonetheless, the ARET condition was still effective in significantly ameliorating approach behavior, similar to a real spider, a finding which questions this differential avoidance hypothesis.

Another limitation is that the control group was only exposed to the BAT at pre-test (T1) and follow-up (T3), but not at post-treatment (T2) unlike the intervention groups which completed the BAT thrice. Thus, the waitlisted participants may have “benefited” from the inadvertent exposure effects via the BAT but to a different extent. Thus, we reanalyzed our data by examining differences between treatment and control groups at the second BAT (i.e., delta T2-T1 for the intervention groups vs. delta T3-T1 for WLC) and still found a significant benefit (*p* = 0.025) for the treatment groups over the controls, *d* = 0.68 (see Supplementary material for more details). Moreover, our general pattern of findings is consistent with numerous previous studies indicating that exposure treatment was superior to waitlist on the BAT (Garcia-Palacios et al., 2002; Hoffman et al., 2003), implying that these procedural differences alone may not have accounted for the large effects we obtained on our primary measures. Finally, the gender-disbalanced nature of our sample was also notable, although generally consistent with the pattern that more women report phobic symptoms (Wardenaar et al., 2017). Prevalence studies have found a considerably higher female to male ratio for animal phobias (4:1 in Sweden; Fredrikson et al., 1996), but our gender ratio may be even more skewed than expected. More point prevalence studies for phobias are needed in the Russian context. While future effectiveness and efficacy research is needed, the overall pattern of results suggests that AR solutions may not need to be highly complicated devices and sophisticated graphically to effect clinically and statistically significant change. Our relatively simple smartphone device, whose program was designed by engineering students, was found to be similarly effective to a traditional approach to exposure.

### 7.2 Future research

Our ARET intervention was efficacious, but also had some technical limitations. Future studies could utilize an updated program that allows a graphically improved spider to maintain a greater flexibility of distance from the patient; this program could be tested in a similar design but with a larger and more gender balanced sample. Control groups and treatment groups need to be assessed in equal frequency to avoid confounding explanations (e.g., due to variations in the number of BAT exposures). Our study was conducted at a lab with patients connected to skin conductance devices as opposed to a more naturalistic setting with fewer selection criteria. Future effectiveness studies could be conducted in a typical clinic setting with more representative clients. Such effectiveness studies may add flexibility to the treatment procedure (e.g., avoiding preset distances in IVET) and separate staff roles for assessment and treatment. We also utilized a one session treatment protocol, although multi-session treatments for animal phobias may also be differentially beneficial (Hemyari et al., 2020). Given that the program is fairly simple, it may be disseminated more broadly to make it accessible to clinicians that may have less access to expensive AR or VR equipment (please contact TS, co-author, for the program details). Future studies should also focus on testing the tool as a self-help treatment without therapist guidance (see Zimmer et al., 2021) or as an adjunct to other more traditional treatments.

## 8 Conclusion

The current study tested a novel smartphone powered augmented reality intervention for spider phobia, comparing it to more traditional *in vivo* exposure therapy and a waitlist control group. Both treatment groups showed statistically significant and clinically meaningful improvements in behavioral approach at post-test that were maintained at 1-month follow- up, compared to the wait-listed group. Moreover, the treatment groups also showed significant improvements in subjective symptom report at 1-month follow up for the SPQ. Although the AR program could benefit from additional refinement, given its utility and potential accessibility, these promising findings suggest that future evaluation research could be conducted in therapy settings with minimal resources. We hope that such relatively simple AR programs can be made accessible to clinicians in various settings, including those in resource deprived clinics, such as in some developing countries (see also Adu et al., 2021).

## Data availability statement

The raw data supporting the conclusions of this article will be made available by the authors, without undue reservation.

## Ethics statement

The studies involving humans were approved by the Institutional Review Board of the National Research University Higher School of Economics. The studies were conducted in accordance with the local legislation and institutional requirements. The participants provided their written informed consent to participate in this study.

## Author contributions

TJ was the principal investigator, contributed to the conceptualization of the study and the design, coordination of the authors, and led the writing of the manuscript text. SZ-P and YKr were involved in the study design, data collection, participant coordination, experiment implementation as therapists, and writing of the method section. VK and A-RM contributed to the conceptualization of the study, study design, questionnaire development, data collection and analysis, writing of the manuscript. TS contributed to the conceptualization of the study, the supervision of NS and AS, preparation of the section on the AR device. IS contributed to questionnaire development and translation, data collection, laboratory care of spider, and experiment implementation as a therapist. NB contributed to participant assignment, experiment implementation as a therapist, and writing of the method section. PA was involved in preparation of the questionnaires, co-authoring the introduction and method. NS and AS contributed to the development of the AR device used. AD was involved in the experiment implementation as a therapist. YKo assisted with supervising therapists and provided conceptual feedback on the manuscript draft. All authors revised the manuscript for important intellectual content. All authors gave final approval of the version to be published.

## Footnotes

^1^ https://github.com/anskuratov/ar-mr-therapy
