# Supplementary material for: The efficacy of augmented reality exposure therapy in the treatment of spider phobia—a randomized controlled trial
Source: Front Psychol. 2024 Feb 19;15:1214125. doi: 10.3389/fpsyg.2024.1214125 (PMC10911123; doi:10.3389/fpsyg.2024.1214125)
Supplement: Supplementary file 1 [file Data_Sheet_1.pdf]

Supplementary Material

The Efficacy of Augmented Reality Exposure Therapy in the Treatment of Spider Phobia – A Randomized Controlled Trial

Tomas Jurcik*[1], Svetlana Zaremba-Pike[1], Vladimir Kosonogov[2], Abdul-Raheem Mohammed[3], Yulia Krasavtseva[4,5,6], Tadamasa Sawada[1,7,8,9], Irina Samarina[10], Nilufar Buranova[6], Peter Adu[11], Nikita Sergeev[12], Andrei Skuratov[12], Anastasia Demchenko[6], Yakov Kochetkov[6]

[1]School of Psychology, HSE University, Moscow, Russia,

[2]HSE University, Russia,

[3]Department of Social and Behavioral Change, School of Public Health, University for Development Studies, Tamale, Ghana,

[4]Department of Pedagogy and Medical Psychology, Sechenov University, Moscow, Russia,

[5]Department of Psychology, Lomonosov MSU, Moscow, Russia,

[6]Center for Cognitive Therapy, Moscow, Russia,

[7]Department of Psychology, Russian-Armenian (Slavonic) University, Yerevan, Armenia,

[8]Akian College of Science and Engineering, American University of Armenia, Yerevan, Armenia,

[9]European University of Armenia, Yerevan, Armenia,

[10]Department of Healthcare, P. B. Gannushkin Moscow Clinical Psychiatric Hospital No. 4, Russia,

[11]Wellington Faculty of Health, Victoria University of Wellington, Wellington, New Zealand,

[12]Department of Computer Engineering, HSE University, Moscow, Russia.

*** Correspondence:** Tomas Jurcik, dr.tomas.jurcik@gmail.com

More detailed information on the exposure protocol, Augmented Reality (AR) device, supplemental measures and post-hoc analyses are provided here. The authors can be contacted for additional information.

1. Supplemental Measures

Additional measures assessing AR system usability (Simulator Sickness Questionnaire; SSQ, Kennedy, 1993; iGroup Presence Questionnaire IPQ; Panahi et al., 2009; System Usability Scale; SUS; Brooke, 1996) are described below. Three measures assessing different domains of using technology were used in the present study. That is, the side effects, sense of engagement, and attitude towards using technologically related devices were measured to ensure participants comfortability for using devices used in this study.

1.1 Simulator Sickness Questionnaire (SSQ; Kennedy et al., 1993).

Cybersickness was assessed with the SSQ. This scale consists of 16 items listing the side effects associated with stimulation in the virtual fields. Responses are rated on a 4-point scale (0= none; 3=severe). Examples of items on the scale are: “headache” and “fatigue”. The scale is divided into three non-mutually exclusive sections denoting symptoms of nausea (N), oculomotor disturbance (O), and disorientation (D). In terms of scoring, each category is summed up and then multiplied by a constant scaling factor. A total score on the scale is the sum of scores from the three categories. Hence, scores are interpreted as: negligible (< 5), minimal (5 – 10), significant (10 – 15), and concerning (15 – 20) symptoms, and scores above 20 are considered a bad intervention (Bimberg et al., 2020). The scale yielded very good internal consistency in the current scale (α = .87). Our current scores (M=5.89; SD=5.49) are suggestive of minimal symptoms related to Cybersickness.

1.2 iGroup Presence Questionnaire (IPQ; Panahi et al., 2009).

Sense of presence experienced in a virtual environment (VE) was measured with igroup Presence Questionnaire (IPQ). This 14-item questionnaire is a self-reported measure assessing three sub variables in a VE: spatial presence (the sense of being physically present in the VE; sample item: “I felt present in the virtual space”); Involvement (measuring the attention devoted to the VE and the involvement experienced; sample item: “I was not aware of my real environment”); experienced realism (measuring the subjective experience of realism in the VE; sample item: “How real did the virtual world seem to you”). All questions were presented in a seven-point Likert scale format using different ratings (e.g., spatial presence was rated on −3=fully disagree; 3=fully agree), the possible overall value range being from −42 to 42. The total Cronbach’s alpha for the full scale was somewhat low (α = .63). In our study no outliers were detected (± 3 SDs; M= −1.74, SD = 10.18, min = −17, max = 22). Our findings (M=-1.74, SD=10.18) suggest that participants may not have agreed that the AR spider felt present to them, but there was a considerable amount of variability, which may partly explain treatment efficacy (see also SUS below, and results).

1.3 System Usability Scale (SUS; Brooke, 1996).

Usability of the virtual reality software was assessed with the SUS. This scale measures one’s attitudes regarding using either website, software, or human-machine systems. This is a 10-item self-report scale measured on a 5-point Likert scale (1=strongly disagree; 5=strongly agree). Examples of items on the scale include: “I found the system very cumbersome to use”

and “I felt very confident using the system.” Participants' scores are converted to a scale from 0 to 4, added together and multiplied by 2.5 to convert the original scores to 0-100. A score of 68 is considered above average and a score below 68 is regarded as below average on the scale (Brooke, 1996). The Cronbach’s alpha for the scale in our study was somewhat low (α = .62). However, no outliers were detected (± 3 *SD*s, min = 45, max = 95), and overall, our findings (*M* = 73.64, *SD* = 12.88) suggest that the system had above average usability.

2. AR device

The AR device used in this study showed a 3D computer-graphics image of a spider superimposed on a photographic image of a scene "out there" (Figure S1). The device (Figure S2) was a head-mounted display that was composed of a smart phone (Lenovo Phab 2 Pro) mounted within a wearable stereoscope (VR head-set). This phone was developed specifically for AR applications (Google Tango). It was equipped with a photo camera for capturing a photographic image of the scene and with a 3D scanner for capturing a depth distribution of the scene. A special Android app, which was developed for this study, was installed in the phone. This app recovered the 3D surfaces in the scene based on a depth distribution. Once this was done, a 3D computer-graphics image of a spider was superimposed on the photographic image, making the spider appear to be on the surface in the scene. The image with the spider was shown on the LCD screen of the smartphone. The CPU of the phone performed this entire process in nearly "real-time". A participant wore the head-set and saw the LCD screen of the phone. The program of this AR app was uploaded to GitHub (https://github.com/anskuratov/ar-mr-therapy).

The 3D model of the spider used in the AR app was modeled after the female wasp spider (Argiope bruennichi), which is common in Russia. The size of the model was about 10 cm, including its legs. It was larger than the real wasp spider. The model had a simplified structure of this spider. It had 34 joints, 3 for each leg, 3 for each pedipalp, 1 for each chelicera, and 2 for the cephalothorax and abdomen. The model could emulate the normal actions of the spider. The AR app could also show a 3D computer-graphics images of a rat (bald/hairless rat, Figure S3A) and a snake (Vipera Nikolskii, Figure S3B) superimposed on the photographic image. The rat and the snake were not used in our study (see Sergeev & Skuratov, 2018 for more details).

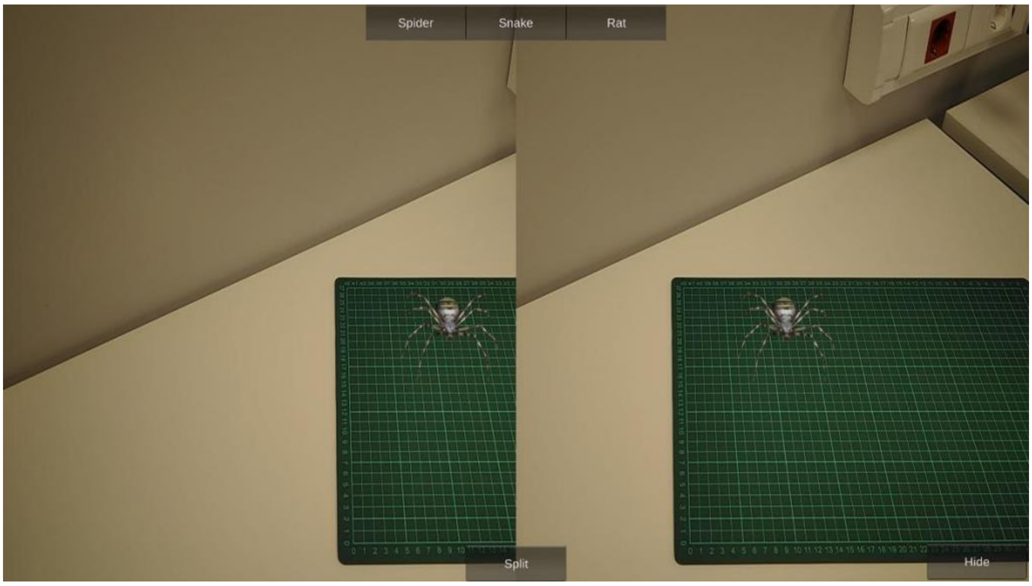

Figure S1. A screenshot of the AR app used in this study. The screen shows a photographic image of a scene "out there" with a 3D computer-graphics image of a spider superimposed. The 3D model of the spider was modeled after the female wasp spider (Argiope bruennichi). This image was shown on the LCD screen of the AR device and the left and right halves of the screen were viewed individually by the two eyes of a participant by using a stereoscope.

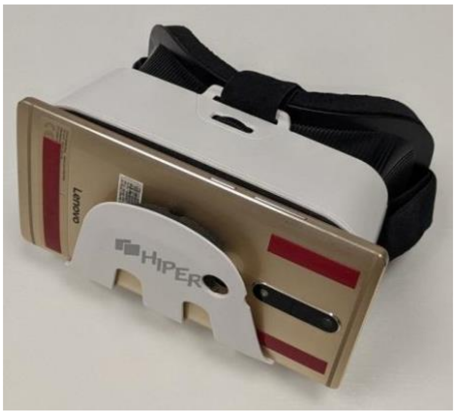

Figure S2. The AR device used in this study. The device was composed of a smart phone (Lenovo Phab 2 Pro) mounted within a wearable stereoscope (VR head-set). The phone was equipped with the photo camera used to capture a photographic image of the scene. The phone was also equipped with an infra-red (IR) projector and the IR camera of the 3D scanner used to capture a depth distribution of the scene.

A B
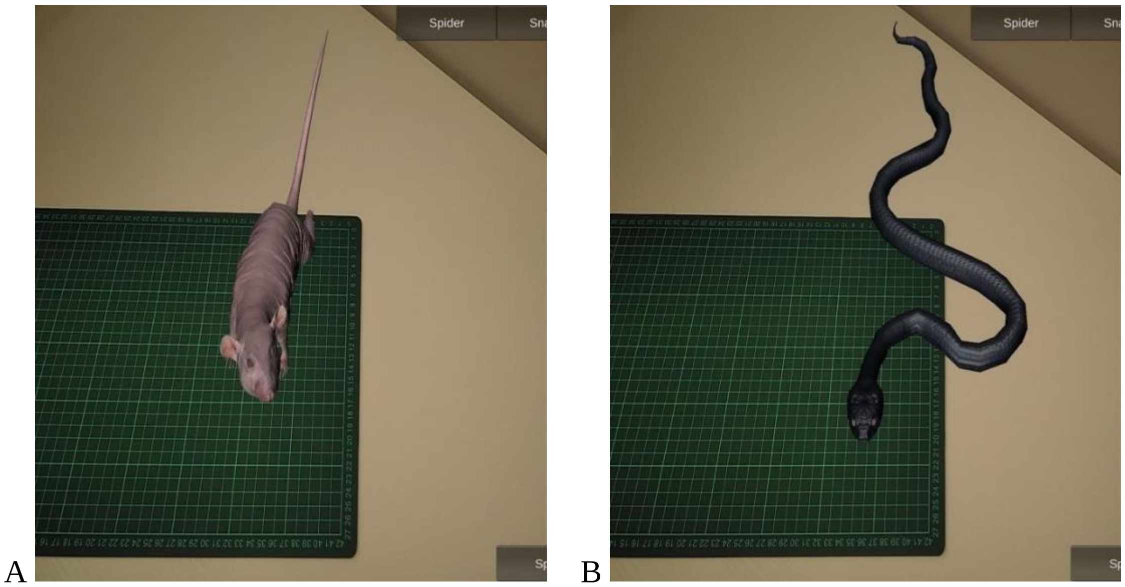

Figure S3. 3D computer-graphics images of (A) a rat (bald/hairless rat) and (B) a snake (Vipera Nikolskii) superimposed on a photographic image of a scene “out there” using this study's AR app.

The 3D scanner of the smartphone measured a depth distribution of a scene using the *time-of-flight* method. The scanner was composed of an infra-red (IR) projector and an IR camera with a resolution of 224 × 172 pixels. The IR projector emitted pulses of invisible IR light and the IR camera detected the reflection of the light from the surface of the scene. Note that the IR light emitted from the projector traveled to the surface and was reflected from the

surface to the IR camera with the speed of light, but there was still a very-short delay between the emission and the detection of the light. The scanner used this delay to compute the distance to the surface for each pixel. The IR camera also measured the intensity of the visible light in each pixel. The AR app recovered the surface and the illuminant condition of the scene from depth and intensity distributions by making use of Google Tango SDK.

The 3D model of the spider, the recovered surface in the scene, and the photographic image of the scene were integrated in the Unity game engine (Unity Technologies). The orientation of the spider was set so that the spider appeared to be on the surface at a position specified by an experimenter. The rendered image of the spider was superimposed on the photographic image. Two image segments (1248 × 1280 pixels) that were taken from the superimposed image were shown on the left and right halves of the LCD screen (see Farshchi et al., 2021 for more details). A participant viewed the two halves of the screen binocularly with the stereoscope and fused the retinal mages of the halves. The visual angle of each image segment was 39° × 40° from the camera and the visual angle of each half of the screen subtended 58° × 59° (6.9 cm × 14.2 cm) from the eyes of the participant.

The refresh-rate of the screen was 20 Hz and there was a delay of 150 msec between the photo camera capturing a scene and the LCD screen showing the pair of images with a spider. The refresh-rate (20 Hz) and the delay (150 msec) should be sufficient for a *subjective* experience with the AR device that was used in this study (see Chen & Thropp, 2007; Cummings & Bailenson, 2016 for reviews).

3. Additional results

3.1 Additional post-hoc analysis on Skin Conductance Response (SCR)

We examined changes over time for the intervention groups. Specifically, SCR in the ARET group did not decrease from pre-test to post-test, $t(19) = 0.26$, $p = .795$, $d = 0.06$, but then it decreased from post-test to 1-month follow up, $t(19) = 3.15$, $p = .005$, $d = 0.70$. In contrast, for the IVET group SCR decreased from pre-test to post-test, $t(17) = 3.06$, $p = .007$, $d = 0.72$ and was maintained from post-test to 1-month follow up, $t(17) = 0.83$, $p = .42$, $d = 0.20$.

When the ARET and IVET groups were compared over time DeltaSCR (post-pretest), SCR decreased more in the IVET group (M = −0.65, SD = 0.91) than in the ARET (M = -0.04, SD = 0.73), $t(36) = 2.29$, $p = .028$, $d = 0.75$. On the contrary, SCR (1 month follow up minus post-test) marginally decreased more in the ARET ($M = -0.60$, $SD = 0.85$) than in the IVET group ($M = -0.15$, $SD = 0.76$), $t(36) = 1.71$, $p = .096$, $d = 0.55$.

3.2 Additional post-hoc analysis on Behaviour Approach Test (BAT)

Due to logistical issues the BAT was performed at pre-test (T1), post-test (T2) and 1-month follow up (T3) for the intervention groups (ARET and IVET) but only at T1 and T3 for the Waitlist Control (WLC) groups. In order to partly account for this potential confound, we examined whether the WLC group improved at T3-T1 (i.e., the second BAT for the controls) to the same extent as the intervention groups did by T2-T1 (i.e., second BAT for the intervention groups) or whether the treatment groups, as expected, would have outperformed WLC by the second BAT.

Thus, the intervention groups combined (ARET+IVET) scored higher at the second compared to the first BAT, *M*([T2-T1])=2.76, SD=2.45, compared to the WLC, *M*(T3-T1), *M*=1.24, *SD*=1.75, *t*(53)=2.31, *p*=0.025, *d*=0.68. This finding supports our conclusions that the treatment groups outperformed the WLC despite discrepancies in the number of BATs conducted.

3.3 Additional post-hoc analysis on Fear of Spiders Questionnaire (FSQ2)

Particularly, FSQ in the ARET group decreased from pre-test to post-test, $t(19) = 3.17$, $p = .005$, $d = 0.71$, but did not change from post-test to 1-month follow up, $t(19) = 0.78$, $p = .445$, $d = 0.18$. Similarly, for the IVET group, FSQ decreased from pre-test to post-test, $t(17) = 2.87$, $p = .011$, $d = 0.68$ and gains were retained from post-test to 1-month follow up, $t(17) = 0.81$, $p = .430$, $d = 0.19$. DeltaFSQ (post-test–pre-test) did not differ between ARET and IVET, $t(36) = 0.08$, $p = .94$. Similarly, Delta FSQ (follow-up-post-test) did not differ between the two treatment groups, $t(36) = 0.09$, $p = .93$.

3.4 Additional post-hoc analysis on Generalized Anxiety Disorder symptoms (GAD-7)

More specifically, the GAD-7 in the ARET group did not decrease from pre-test to post-test, $t(19) = 0.55$, $p = .585$, $d = 0.13$, or from post-test to 1-month follow up, $t(19) = 1.04$, $p = .314$, $d = 0.24$. While GAD scores in the IVET group did not decrease from pre-test to post-test, $t(17) = 0.52$, $p = .608$, $d = 0.13$, they marginally increased from post-test to 1-month follow-up, $t(17) = 1.90$, $p = .074$, $d = 0.45$. DeltaGAD (post-test minus pre-test) did not differ between ARET and IVET, $t(36) = 0.65$, $p = .425$. However, DeltaGAD (1-month follow-up minus post-test) was larger for IVET in comparison to ARET, $t(36) = 2.43$, $p = .020$.

3.5 Additional post-hoc analysis on Physical Health Questionnaire (PHQ)

Particularly, PHQ in the ARET group remained stable from pre-test to post-test, $t(19) = 0.20$, $p = .846$, $d = 0.04$, and from post-test to 1-month follow-up, $t(19) = 0.486$, $p = .314$, $d = 0.17$. PHQ in the IVET group showed no change from pre-test to post-test, $t(17) = 1.50$, $p = .152$, $d = 0.36$, or from post-test to 1-month follow-up, $t(17) = 0.60$, $p = .558$, $d = 0.14$. DeltaPHQ (post-test minus pre-test) also did not differ between the two treatment conditions, $t(36) = 2.46$, $p = .126$. Similarly, DeltaPHQ (1-month follow-up minus post-test) did not differ between the two treatment groups, $t(36) = 0.12$, $p = .736$. This divergent pattern indicates that our general measures were not affected by the treatment in the same way as our phobia specific measures.

References

Bimberg, P., Weissker, T., & Kulik, A. (2020). On the usage of the simulator sickness questionnaire for virtual reality research. In *2020 IEEE conference on virtual reality and 3D user interfaces abstracts and workshops (VRW)* (pp. 464–467). IEEE. https://doi.org/10.1109/VRW50115.2020.00098

Brooke, J. (1996). SUS-A quick and dirty usability scale. *Usability Evaluation in Industry, 189*(194), 4–7.

Chen, J. Y., & Thropp, J. E. (2007). Review of low frame rate effects on human performance. *IEEE Transactions on Systems, Man, and Cybernetics-Part A: Systems and Humans, 37*(6), 1063–1076. https://doi.org/10.1109/TSMCA.2007.904779

Cummings, J. J., & Bailenson, J. N. (2016). How immersive is enough? A meta-analysis of the effect of immersive technology on user presence. *Media Psychology, 19*(2), 272–309. https://doi.org/10.1080/15213269.2015.1015740

Farshchi, M., Kiba, A., & Sawada, T. (2021). Seeing our 3D world while only viewing contour-drawings. *PloS one*, *16*(1), e0242581. https://doi.org/10.1371/journal.pone.0242581

Kennedy, R. S., Lane, N. E., Berbaum, K. S., & Lilienthal, M. G. (1993). Simulator sickness questionnaire: An enhanced method for quantifying simulator sickness. *The International Journal of Aviation Psychology, 3*(3), 203–220. https://doi.org/10.1207/s15327108ijap0303_3

Panahi, S. M., Fathi, A. A., Azad, F. P., & Montazer, G. A. (2009). Reliability and Validity of Igroup Presence Questionnaire (IPQ). *International Journal of Behavioral Sciences*, *3*, 27–34.

Sergeev, N., & Skuratov, A. (2018). Development of a gamified mixed reality system: Towards an exposure therapy for animal phobia [Unpublished undergraduate thesis]. HSE University.
